# Tests for Sugar in the Urine

**Published:** 1905-12-16

**Authors:** 


### TESTS FOR SUGAR IN THE URINE.

"Many a candidate for life insurance, otherwise sound in all respects, has been rejected solely because his urine gave a questionable reaction with Fehling's solution." With these words Mr. L. C. S. Broughton [1] opens a paper dealing with the relative merits and deficiencies of a number of tests for sugar in the urine. The statement is perfectly just, and, as the author observes, gives much food for thought in view of the acknowledged fact that there are numerous substances from time to time present in the urine, and, so far as is known, of no ominous significance, which are nevertheless capable of reducing the copper salts commonly employed to detect the presence of glucose.

It is still a debated question whether or not the appearance of sugar in the urine is compatible with the healthy physiological functions of the various organs occupied with the metabolism of carbohydrates. Each point of view has numerous supporters: thus Pavy, Bence-Jones, and Halliburton are of opinion that physiological glycosuria is a fact to be reckoned with, while such authorities as Friedländer and Sir George Johnson deny the existence of such a phenomenon. The tendency of recent research is to admit the possibility of glycosuria in the perfectly healthy. The proportion of sugar which is to be admitted under the title of a physiological quantity is, however, very small; it varies from .001 per cent. (Lohnstein) to .05 per cent. (Pavy).

Glucose is a very powerful reducing agent, as is evidenced by the fact that it is capable of reducing some salts of copper, cobalt, bismuth, gold, iron, lead, mercury, nickel, silver, and also many aniline dyes, such as gentian-violet, indigo-carmine, methylene blue, picric acid, and safranin. There is, however, this serious defect in all the reduction tests for sugar—namely, that in all of them the oxidiser requires for its activity the addition of a caustic alkali. The author points out that undoubtedly one ought to reckon on the possible activity of the alkali, whereas little or nothing appears to be known on this head.

Mr. Broughton classifies as follows the various tests to which attention is given:—

1. Reduction tests:—

*Metallic.*

Fehling's solution.
Nylander's solution.
Trommer's test.
Rubner's test.

*Non-metallic.*

Indigo-carmine.
Orthonitrophenylpropionic acid.
Safranin.
Methylene blue.
Picric acid.

2. Non-reduction tests:—

Moore's test.
Fermentation test.
Phenylhydrazine test.

Of all this imposing array there are four only which appear to have gained the author's confidence. These four are Fehling's solution, Nylander's solution, the fermentation test, and the phenylhydrazine test. The following details of their respective application and the fallacies that attend their use are of considerable practical importance.

Prior to the examination of any specimen of urine for sugar, it is essential that any albumin present in the specimen should be removed, for the presence of albumin will vitiate every test for sugar except the fermentation test. This removal is easily achieved by the addition of a little acetic acid, followed by boiling and filtering. Further, all turbid urines, especially when the turbidity depends upon uratic deposits, should be filtered before any test for sugar is applied.

*Fehling's test.*—The method recommended as obtaining the best results with this solution is to boil an inch of the reagent in a test-tube. If the solution keeps clear, fifteen drops of urine are to be added and the mixture heated again, but kept below the boiling-point. A positive result is indicated by a brick-red precipitate of anhydrous oxide of copper, or by a yellow precipitate of the hydrated oxide. If the solution of Fehling's solution becomes turbid on being boiled, it is well to discard the sample, for the turbidity is evidence of a chemical decomposition which may vitiate the test. The solution will keep for a great length of time if its ingredients are pure, but, generally speaking, it is wise to keep the copper and the soda solutions in separate bottles until the mixture is required.

Regarding the fallacies attending tests that involve the reduction of copper salts. There are several substances, normal constituents of urine, which are capable of effecting this. Of these the chief are uric acid and urates, kreatinin and glycuronic acid. But, generally speaking, the reducing capacity of these substances is sufficiently small for them to require prolonged boiling before evidence of reduction appears. In the case of sugar, on the other hand, reduction is evident before the boiling-point is reached; indeed, it will take place in the cold, but only after a lengthened interval. In this earlier appearance of reduction in the case of sugar lies the explanation of the advice that the mixture of urine and Fehling's solution should not be boiled.

Ammoniacal urine should not be tested with Fehling's solution, for if the quantity of sugar present be small, the only result will be a slight fading of colour, which is easily overlooked.

It is important not to add too much urine, for in such a case the caustic alkali may lead to the precipitation of earthy phosphates which may simulate a reduction product.

Occasionally one meets with cases in which the specimen, on being cooled, appears as a turbid fluid of a turquoise or greenish-yellow colour. For such a case the other three methods are useful corroboratives.

*Nylander's test.*—This is described by the author as an excellent test capable of detecting .1 per cent. of sugar. The reagent is made as follows. With the aid of gentle heat a solution is made according to the accompanying prescription.

| | |
|---|---|
| Bismuth subnitrate ... ... | 2 grammes |
| Rochelle salt ... ... ... | 4 ,, |
| Caustic soda ... ... ... | 10 ,, |
| Distilled water ... ... ... | 100 c.c. |

This forms a colourless liquid which will keep indefinitely. The test is applied by boiling together in a test-tube, for two minutes, three drachms of urine and twenty drops of the reagent. A positive reaction is shown by a blackening of the mixture due to the formation of the sulphide of bismuth.

The *fermentation test* affords complete proof of the presence of sugar. The fallacies that attend its use are a possible inactivity of the yeast employed, and the presence of starch in the yeast, as commonly happens in the commercial product. In consequence of these possibilities the test is inconclusive unless control experiments are simultaneously conducted. The test is rather too complicated for routine work; moreover, it is unsatisfactory when the percentage of sugar present falls below .3 per cent.

The *phenylhydrazine test* is "the most serviceable and one of the most delicate tests we have." It is carried out as follows: five grains of phenylhydrazine hydrochloride and ten grains of sodium acetate are added to half an ounce of urine; the whole is placed in a water-bath, and boiled for half an hour. On being cooled, the mixture, if it contains sugar, will deposit fans, stars, and sheaves of phenylglucosazone crystals, which are easily identified under a low power of the microscope. This test will detect a quantity of sugar so small as to be by some considered physiological; but in such a case the mass of crystals is very insignificant.

In addition to the above fallacies it is well to remember that there is a large number of drugs known to affect the action of Fehling's solution. Such, for instance, are alcohol in large quantity, arsenic, aspirin, chloral hydrate and butyl-chloral hydrate, camphor, chloroform, carbolic acid, morphine, salol and salicylic acid, sulphonal, and many coal-tar products. Nylander's solution is also influenced by arsenic, carbolic acid, iodides and mercury, quinine, rhubarb, salicylic acid, sulphur, and turpentine. A positive reaction is also produced by melanin if that be present in the urine.

[1] Birmingham Med. Rev., October 1905.

